# *DHH-RHEBL1* fusion transcript: a novel recurrent feature in the new landscape of pediatric *CBFA2T3-GLIS2*-positive acute myeloid leukemia

**DOI:** 10.18632/oncotarget.1280

**Published:** 2013-09-07

**Authors:** Riccardo Masetti, Marco Togni, Annalisa Astolfi, Martina Pigazzi, Elena Manara, Valentina Indio, Carmelo Rizzari, Sergio Rutella, Giuseppe Basso, Andrea Pession, Franco Locatelli

**Affiliations:** ^1^ Department of Pediatrics, “Lalla Seràgnoli”, Hematology-Oncology Unit, University of Bologna, Italy; ^2^ Giorgio Prodi Cancer Research Center, University of Bologna, Bologna, Italy; ^3^ Department of Woman and Child Health, Laboratory of Hematology-Oncology, University of Padova, Padova, Italy; ^4^ Biocomputing Group, Department of Biological, Geological and Environmental Sciences (BiGeA), University of Bologna; ^5^ Department of Pediatrics, San Gerardo Hospital, University of Milano-Bicocca, Monza, Italy; ^6^ Department of Pediatric Hematology-Oncology, IRCCS Ospedale Bambino Gesù, Rome, Italy; ^7^ University of Pavia, Pavia, Italy

**Keywords:** pediatric acute myeloid leukemia, cytogenetically normal acute myeloid leukemia, whole-transcriptome massively parallel sequencing, CBFA2T3-GLIS2 fusion transcript, DHH-RHEBL1 fusion transcript

## Abstract

Childhood Acute Myeloid Leukemia (AML) is a clinically and genetically heterogeneous malignant disease. Despite improvements in outcome over the past decades, the current survival rate still is approximately 60-70%. Cytogenetic, recurrent genetic abnormalities and early response to induction treatment are the main factors predicting clinical outcome. While the majority of children carry recurrent chromosomal translocations, 20% of patients do not show any recognizable cytogenetic alteration and are defined to have cytogenetically normal AML (CN-AML). This subset of patients is characterized by a significant heterogeneity in clinical outcome, which is influenced by factors only recently started to be identified. In this respect, genome-wide analyses have been used with the aim of defining the full array of genetic lesions in CN-AML. Recently, through whole-transcriptome massively parallel sequencing of seven cases of pediatric CN-AML, we identified a novel recurrent *CBFA2T3-GLIS2* fusion, predicting poorer outcome. However, since the expression of *CBFA2T3-GLIS2* fusion in mice is not sufficient for leukemogenesis, we speculated that further unknown abnormalities could contribute to both cancer transformation and response to treatment. Thus, we analyzed, by whole-transcriptome sequencing, 4 CBFA2T3-GLIS2-positive patients, as well as 4 CN-AML patients. We identified a new fusion transcript in the *CBFA2T3-GLIS2* -positive patients, involving *Desert Hedgehog* (*DHH*), a member of Hedgehog family, and *Ras Homologue Enrich in Brain Like 1* (*RHEBL1*), a gene coding for a small GTPase of the Ras family. Through the screening of a validation cohort of 55 additional pediatric AML patients, we globally detected *DHH-RHEBL1* fusion in 8 out of 20 (40%) *CBFA2T3-GLIS2-* rearranged patients. Gene expression analysis performed on RNA-seq data revealed that *DHH-RHEBL1* –positive patients exhibited a specific signature. These 8 patients had an 8-year overall survival worse than that of the remaining 12 *CBFA2T3-GLIS2-* rearranged patients not harboring *DHH-RHEBL1* fusion (25% *vs* 55%, respectively, *P* =0.1). Taken together, these findings are unprecedented and indicate that the *DHH-RHEBL1* fusion transcript is a novel recurrent feature in the changing landscape of *CBFA2T3-GLIS2* -positive childhood AML. Moreover, it could be instrumental in the identification of a subgroup of *CBFA2T3-GLIS2* -positive patients with a very poor outcome.

## INTRODUCTION

Childhood acute myeloid leukemia (AML) encompasses a heterogeneous group of malignancies, with great variability in terms of response to therapy. While the majority of patients harbor recurrent chromosomal translocations, almost 20% of childhood AML do not show any recognizable cytogenetic alteration and are defined as cytogenetically-normal (CN-AML) [1]. Recently, an increasing list of molecular markers with prognostics significance has been identified in adult CN-AML [2-8]. However, these alterations are barely detected in pediatric CN-AML [9-11] and there is a considerable proportion of children with CN-AML in whom no genetic abnormality can be unveiled.

In a recent study, we performed whole-transcriptome massively parallel sequencing of 7 cases of pediatric CN-AML; in 3 of them, we identified a novel recurrent fusion transcript involving *CBFA2T3* and *GLIS2* genes. We then extended the analysis to a larger cohort (N=230) and this novel fusion was detected in 20 out of the 237 pediatric CN-AML cases analyzed (8.4%). The 5-year event-free survival of the 20 positive patients was significantly worse than that of the 217 pediatric CN-AML patients lacking the translocation (27.4%vs 59.6%; *P=* 0.01), demonstrating that *CBFA2T3-GLIS2* fusion transcript is a novel common feature of pediatric CN-AML predicting poorer outcome [12].

Despite this evidence in human AML, expression of *CBFA2T3-GLIS2* fusion is not sufficient to foster leukemia development in mice, this suggesting that the fusion protein *per se* may not promote leukemogenesis [13,14]. Starting from this observation, we reasoned that additional lesions can concur to leukemia development in children harboring *CBFA2T3-GLIS2* fusion transcript. Using whole-transcriptome sequencing, we identified a novel fusion transcript that is recurrent in *CBFA2T3-GLIS2*-positive patients and is instrumental for the identification of a subset of *CBFA2T3-GLIS2*-rearranged patients characterized by an even poorer outcome.

## RESULTS

### Identification of DHH-RHEBL1 fusion transcript in pediatric CBFA2T3-GLIS2-positive AML patients by whole-transcriptome sequencing

**Figure 1:**
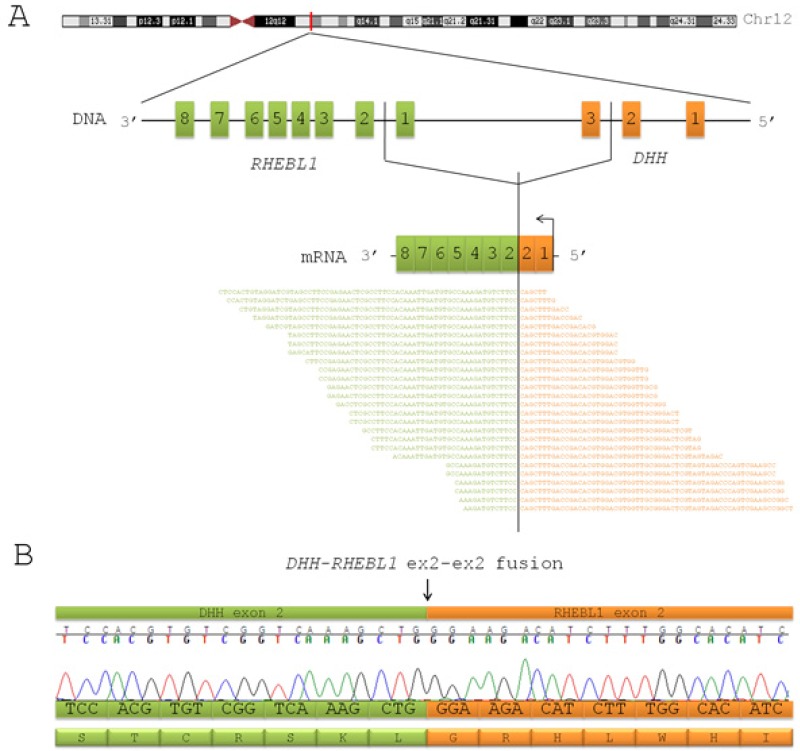
DHH-RHEBL1 is a novel fusion transcript recurrent in pediatric CBFA2T3-GLIS2 positive AML (A) Schematic representation of the fusion between *DHH* and *RHEBL1* identified by means of whole-transcriptome sequencing. The figure shows the position of *DHH* and *RHEBL1* on chromosome 12 and the fusion transcript detected by RNA-seq. The identification of this novel fusion transcript was supported by an average of 11 span and 21 split reads. (B) Sanger sequencing performed in order to validate the detection of the *DHH-RHEBL1* fusion transcript. Electropherogram and predicted sequence of the fusion protein are shown. The black arrow indicates the fusion breakpoint.

Blasts from 8 pediatric patients with AML were analyzed by means of whole-transcriptome massively parallel sequencing. Four of them had CN-AML (CN#21, CN#22, CN#23, CN#24), and 4 harbored *CBFA2T3-GLIS2* fusion transcript (#1, #3, #13, #17). Among the latter, 2 had already been reported in our previous study [12]. Further analysis on RNA-seq data revealed the presence of a new recurrent fusion transcript in 2 out of 4 *CBFA2T3-GLIS2*-positive patients. This novel fusion transcript is the result of a read-through that combines at least part of one exon with each of two distinct (parent) genes that are adjacent on the same chromosome in the same orientation [15]. In particular, this transcript involves *DHH*, a member of the Hedgehog family [16], and *RHEBL1*, a gene coding for a small GTPase of the Ras family which regulates a wide variety of cellular functions, including cell growth, differentiation, and transformation [17]. Both genes are contiguously localized on the reverse strand of chromosome 12 (Figure 1A) and, although the mechanism that leads to generation of read-through fusion transcripts remains obscure [18], RT-PCR analysis and Sanger sequencing confirmed that all positive cases harbored the in-frame fusion between exon 2 of *DHH* and exon 2 of *RHEBL1* (Figure 1B).

### DHH-RHEBL1 fusion transcript is recurrent in pediatric CBFA2T3-GLIS2-positive AML

To determine the prevalence of *DHH-RHEBL1* fusion in pediatric AML, we then examined a validation cohort of 55 children with AML. The validation cohort included CN-AML patients (N=24), *CBFA2T3-GLIS2*-positive patients (N=16), patients harboring known cytogenetic/genetic abnormalities (alteration of *MLL, NPM1, FLT3*, t(8;22)(p11;q13), *t*(*9*;*11*)(p22;q23), inv(16)(p13;q22)) (N=12) and normal CD34^+^ hematopoietic stem cells (N=3). The *DHH-RHEBL1* fusion transcript was detected in 6 out of 16 patients carrying the *CBFA2T3-GLIS2* fusion, while it was never found in the other patients with AML, irrespectively of the mutational status, as well as in normal CD34^+^ cells. Thus, considering also the patients of the sequencing cohort, the *DHH-RHEBL1* fusion was globally present in 8 out of 20 (40%) of the *CBFA2T3-GLIS2-*positive patients, this demonstrating that this novel alteration is a common feature of this peculiar subset of childhood AML.

### DHH-RHEBL1–positive patients exhibit a specific gene expression signature and an overexpression of both DHH and RHEBL1

**Figure 2:**
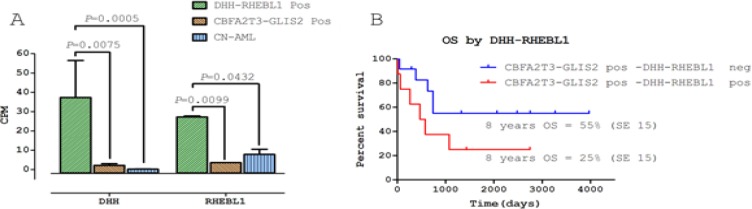
Implications of DHH-RHEBL1 fusion transcript expression (A) Expression levels of *DHH* and *RHEBL1* gene obtained from RNA-seq data in *DHH-RHEBL1* positive patients (N=2), in *CBFA2T3-GLIS2* positive patients (N=2) and in CN-AML patients (N=4). Abbreviations: CPM = count per million, Pos = positive (B) Probability of 8-year overall survival (OS) in *CBFA2T3-GLIS2*-positive children who did or did not harbor the *DHH-RHEBL1* fusion transcript (25%, SE=15 vs 55%, SE=15) (*P*=0.1).

**Figure 3:**
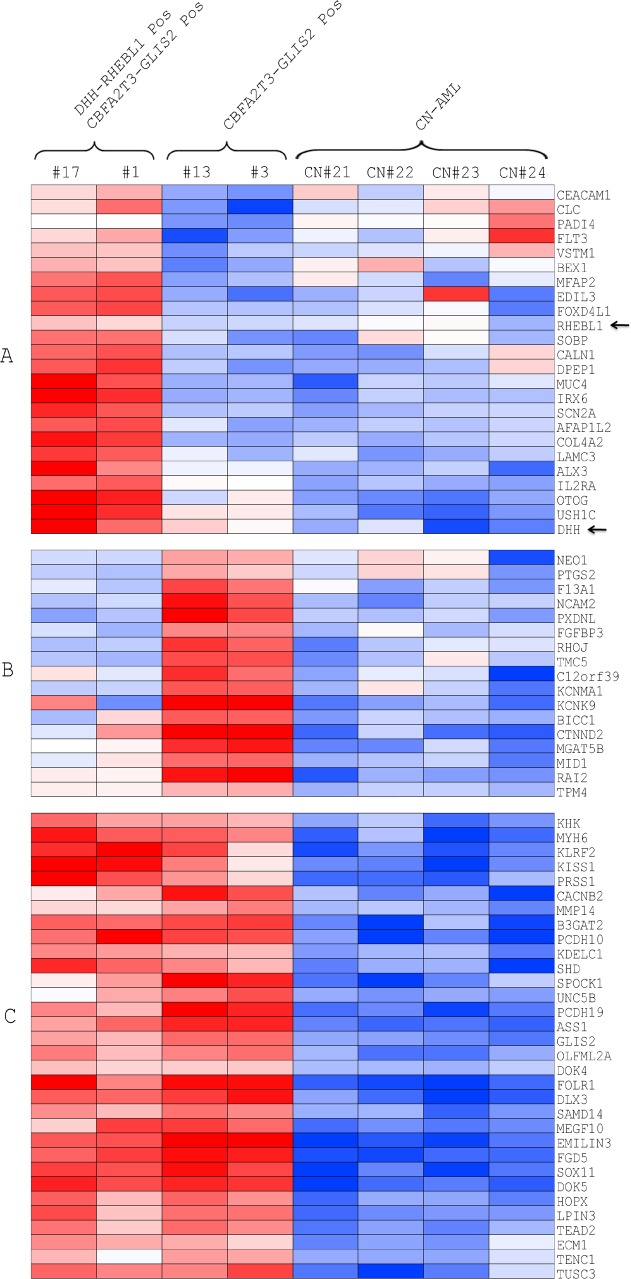
Analysis of gene expression profile of DHH-RHEBL1-positive patients Heatmap of the top 30 differentially expressed genes in *DHH-RHEBL1*-positive patients compared with the patients harboring the *CBFA2T3-GLIS2* fusion only and CN-AML patients. (A) Gene expression signature of patients harboring both *CBFA2T3-GLIS2* and *DHH-RHEBL1* fusion transcript; (B) Gene expression signature of *CBFA2T3-GLIS2*-positive patients; (C) Gene expression signature of children with CN-AML not harboring any detectable fusion transcript. Abbreviation: Pos = positive.

To get insights into the molecular consequences of *DHH-RHEBL1* expression, we performed a gene expression analysis on the RNA-seq data for the 8 patients carrying this fusion transcript. Firstly, we analyzed the expression level of the two genes involved in the fusion transcript and we found that the expression of both *DHH* and *RHEBL1*is significantly enhanced in the *DHH-RHEBL1*-positive patients as compared with patients harboring only *CBFA2T3-GLIS2* fusion (*P*=0.007 and *P*=0.009 respectively) and with the other CN-AML cases (*P*=0.0005 and *P*=0.043, respectively) (Figure 2A). Additionally, *DHH-RHEBL1*-positive patients showed a distinctive gene expression signature both with respect to CBFA2T3-GLIS2-positive patients (518 differentially expressed genes; *P*<0.05), and CN-AML patients (596 differentially expressed genes; *P*<0.05). Interestingly, *DHH-RHEBL1*-positive patients showed higher expression of several genes known to be associated with leukemia occurrence and/or tumor progression, such as *FLT3* [19], *BEX1* [20], *MUC4* [21] and *AFAP1L2* [22] (Figure 3). Finally, we also evaluated whether the presence of *DHH-RHEBL1* fusion transcript influences the outcome of *CBFA2T3-GLIS2*-positive patients. The 8-year overall survival of the 8 patients harboring the *DHH-RHEBL1* fusion transcript was worse than that of the 12 *CBFA2T3-GLIS2-*rearranged patients not harboring the *DHH-RHEBL1* fusion transcript (25% *vs* 55%). Likely due to the small number of patients, this difference failed to achieve statistical significance (*P*=0.1) (Figure 2B).

## DISCUSSION

The last decades have witnessed the identification, through chromosomal analysis techniques or RT-PCR, of fusion genes influencing either proliferation/apoptosis or differentiation potential of AML cells [1-5]. Recently, different studies took advantage of next-generation sequencing approach to identify novel mutations or chromosomal aberrations that escape detection by conventional cytogenetic techniques and characterize pediatric AML, as well as other hematopoietic cancers [12,13,23-25]. Next-generation sequencing, as well as gene expression profiling, are also instrumental to refine the biological peculiarities affecting the risk of treatment failure and to detect crucial pathways potentially ‘druggable’ in both adult and childhood AML [2, 26, 27].

In this regards, we recently demonstrated that *CBFA2T3-GLIS2* fusion transcript is recurrent in pediatric CN-AML and portends a poor outcome [12]. However, experimental evidence demonstrates that expression of this fusion product in mice is not sufficient to promote leukemogenesis [13,14]. This observation prompted us to investigate by whole-transcriptome sequencing a cohort of CN-AML and *CBFA2T3-GLIS2*-positive patients. We were able to identify a novel fusion transcript involving *DHH* and *RHEBL1* genes in 2 out of 4 *CBFA2T3-GLIS2*-positive cases of our sequencing cohort. Extending the analysis to a validation cohort of 55 children, the *DHH-RHEBL1* fusion was globally detected (including the 2 cases in the sequencing cohort) in 8 out of 20 (40%) of the *CBFA2T3-GLIS2-*positive patients, indicating that this novel fusion transcript is recurrent and peculiar to this specific group of pediatric CN-AML. Interestingly, both *DHH* and *RHEBL1* genes have been implicated in a variety of human diseases, including cancer. On the one hand, *DHH* codes for a member of the Hedgehog (HH) signaling pathway, which, similar to other HH ligands, binds to its receptor Patched and leads to the signaling cascade of repressive interactions, culminating into effects on the transcription of target genes. The HH signaling, during embryogenesis, controls cell proliferation, differentiation and tissue morphogenesis [28]. However, it is also well known to have a role in tumors, and the role that HH signaling plays in the growth of tumors can be classified according to how the pathway is activated [29]. These mechanisms include loss-of-function mutations in inhibitory proteins, such as Patched (PTC1), gain-of-function mutations in positive regulators, such as Smoothened (SMO), and overexpression of the HH ligands (Sonic, Indian and Desert Hedgehog), leading to either autocrine or paracrine activation of the pathway and renewal/propagation of cancer stem cells [28]. Recently, with the identification of *CBFA2T3-GLIS2* fusion transcript in pediatric CN-AML, different studies [13,14,23] demonstrated that the presence of this fusion transcript leads to an aberrant activation of the HH signaling due to the ectopic expression of the *GLIS2* transcription factor. Notably, in the present work, we demonstrate that patients harboring the *DHH-RHEBL1* fusion present an overexpression of *DHH* compared to both *CBFA2T3-GLIS2-*positive patients and CN-AML patients (Figure 2A). Considering that overexpression of the HH ligands leads to activation of the HH pathway [28], it is tempting to speculate that overexpression of *DHH* could contribute to the aberrant activation of the HH pathway. On the other hand, the RHEBL1 protein belongs to the Ras family of small GTPases and, similar to other Ras proteins, is a molecular switch that controls a wide variety of cellular functions including cell growth, differentiation and transformation [17]. Previous studies reported that *RHEBL1* could function as an activator of NF-kB [17] and mTOR [30] signaling, both of which are frequently altered in many solid tumors, as well as in leukemias and lymphomas[27, 31-36]. In view of *RHEBL1* over-expression in patients harboring the *DHH-RHEBL1* fusion transcript compared to those harboring only the *CBFA2T3-GLIS2* fusion transcript and to CN-AML children, it will be interesting to investigate more thoroughly its possible role in leukemogenesis.

To define the implications, if any, of *DHH-RHEBL1* fusion transcript expression, we performed an analysis of gene expression on RNA-seq data. Firstly, we demonstrated that patients harboring only the *CBFA2T3-GLIS2* fusion transcript and patients harboring both *CBFA2T3-GLIS2* and *DHH-RHEBL1* fusion transcripts exhibited an overexpression of *GLIS2* compared to CN-AML patients, this finding being consistent with recently published data [13,14,23]. In addition, and more importantly, gene expression profile revealed that *DHH-RHEBL1-positive* patients showed a specific gene expression signature, with 518 and 596 genes being significantly overexpressed when compared to patients harboring only the *CBFA2T3-GLIS2* fusion transcript (*P*<0.05) and CN-AML patients (*P*<0.05), respectively. Interestingly, *DHH-RHEBL1*-positive patients exhibited an enhanced expression of *FLT3*, which has been reported to be constitutively activated and over-expressed in a proportion of both pediatric and adult AML [1,19]. Additionally, also *BEX1*, which is known to be expressed in AML with *MLL* rearrangements [20], was up-regulated. Furthermore, other genes, such as *MUC4* and *AFAP1L2*, associated with different human cancers [21,22], resulted overexpressed in *DHH-RHEBL1*-positive patients. Taken together, these findings indicate that *DHH-RHEBL1*-positive patients exhibit a distinct expression signature compared to both CN-AML patients and to *CBFA2T3-GLIS2*-positive patients (Figure 3), suggesting that the presence of *DHH-RHEBL1* fusion transcript could be important in the definition of a new subgroup among the *CBFA2T3-GLIS2*-positive patients. To assess whether the presence of *DHH-RHEBL1* fusion transcript affects patients' outcome, we estimated the 8-year overall survival of the 8 patients harboring the *DHH-RHEBL1* fusion transcript, finding that it was worse, although not statistically different, than that of the 12 *CBFA2T3-GLIS2-*rearranged patients not harboring *DHH-RHEBL1* fusion (25% *vs* 55%).

While the discovery of gene fusion resulting from chromosomal aberrations, such as translocations, deletion/insertion, inversion, represents the primary objective of gene fusion analyses, whole-transcriptome sequencing aims at unraveling another category of gene fusion that are referred to as read-through fusion transcripts[15,18]. Several studies indicate that these groups of fusion transcripts are widely observed in virtually all samples analyzed, including samples from non-transformed different tissues [37]. Some of these RNA chimeras, however, appear to be restricted to individual tissue types, and a few of these have been observed to be highly expressed in cancers, this observation underpinning the potential functional relevance with respect to cellular differentiation and disease development [38-42]. To the best of our knowledge, *DHH-RHEBL1* is the first read-through fusion transcript reported in a specific subset of pediatric leukemias and its recurrence in *CBFA2T3-GLIS2*-positive patients suggests it could be important for leukemogenesis. In summary, we discovered a novel *DHH-RHEBL1* fusion transcript that is recurrent (40%) in *CBFA2T3-GLIS2*-positive patients only; it characterizes a subset of patients with an even more dismal outcome among the *CBFA2T3-GLIS2*-positive patients. The mechanism(s) through which this fusion transcript promotes leukemogenesis and the possibility of targeting it with pathway-specific compounds remain to be thoroughly investigated.

## METHODS

### Patient samples

After obtaining written informed consent, patient samples analyzed either in the parallel sequencing screening or in the validation cohort were collected from children with newly diagnosed *de novo* AML other than promyelocytic leukemia, enrolled in the Associazione Italiana Ematologia Oncologia Pediatrica (AIEOP) 2002/01 Protocol [43]. Morphological diagnosis and immunophenotypic analysis was centrally reviewed at the laboratory of Pediatric Hematology of the University Hospital in Padova. Chromosome analysis was performed on bone marrow (BM) aspirates using standard laboratory procedures. Karyotypes were reported according to the International System for Human Cytogenetic Nomenclature (ISCN 2005). For fluorescence *in-situ* hybridization (FISH), an *MLL* locus specific (LSI) dual color probe for 11q23 (Abbott-Vysis, Downers Grove, IL) was employed. All the 8 patients of the sequencing cohort were cytogenetically normal, as well as negative for known recurrent genetic abnormalities involving *MLL*, *CBFB*, *NPM1* and *FLT3*.

### Whole-Transcriptome Sequencing and RNA-seq bioinformatics analyses

RNA library construction and whole-transcriptome sequencing has been described previously [12]. In brief, 250-1000 ng of total RNA were used for the synthesis of cDNA libraries with TruSeq RNA Sample Prep Kit v2 (Illumina, San Diego, CA), and sequenced by synthesis at 75bp in paired-end mode on HiScanSQ sequencer (Illumina). Reads were aligned with TopHat2/BowTie2 [44] to the reference human genome hg19/GRCh37. Defuse [45] and Chimerascan [46] packages were used to detect chimeric transcripts from RNA-seq data. Whole-transcriptome massively parallel sequencing in the 8 children with CN-AML yielded an average of 78.4 million mapped reads/patient, thus reaching an average coverage of 34X.

### Gene expression analysis

The mapped reads obtained with TopHat2/BowTie2 pipeline were processed with SAMtools [47] in order to remove the potential optical or PCR duplicate (function “rmdup”) and then the count of the mapped reads for each hg19 gene was performed by applying the Python package “htseq-count” (http://www-huber.embl.de/users/anders/HTSeq/doc/overview.html). Gene annotations were derived from Ensembl Release 70 (January 2013).

The differentially expressed genes were determined with edgeR, a R-bioconductor package suitable for analyzing RNA-seq data [48]. Three different comparisons were performed, corresponding to all the possible couples among the three groups of patients: 1) *DHH-RHEBL1*-positive and *CBFA2T3-GLIS2*-positive (N=2); 2) *DHH-RHEBL1*-negative and *CBFA2T3-GLIS2*-positive (N=2); 3) CN-AML (N=4).

For each comparison, the complete set of genes, with the corresponding mapped reads count, was firstly reduced in order to consider in our analysis only the genes with count-per-million (CPM) > 3 in more than 2 samples. Then, adopting a statistical method based on the negative binomial distribution, the significance of the differences between the normalized reads count was determined for each gene. Differences with *P*<0.05 were considered to be statistically significant.

The Multi Experiment Viewer (MeV) tool (http://www.tm4.org/mev.html) was used to visualize the expression data.

### Screening forDHH-RHEBL1 fusion transcript in the validation cohort

Total RNA was extracted from BM leukemia cells using TRIzol. *DHH-RHEBL1* fusion transcript was detected through RT-PCR and sequenced with the BigDye terminator v3.1 Cycle Sequencing kit (PE Applied Biosystems, Foster City, CA) on an Applied Biosystems 310 analyzer. The RT-PCR was performed at 60°C with the Expand Long Template PCR system (Roche, Mannheim, Germany). Primers used to amplify the full *DHH-RHEBL1* fusion transcript (1359bp) were: forward 5'- AGTAGCAGGTCCTAGACACCCCC -3', reverse 5'- TACCCGTGAAGTCCTGAGGATCT -3'.
